# Resistomycin Inhibits Wnt/β-Catenin Signaling to Induce the Apoptotic Death of Human Colorectal Cancer Cells

**DOI:** 10.3390/md21120622

**Published:** 2023-11-29

**Authors:** Yaoyao Zhu, E Zhang, Huan Gao, Chuangeng Shang, Mengxiong Yin, Mingtao Ma, Yu Liu, Xuanfeng Zhang, Xia Li

**Affiliations:** 1 Marine College, Shandong University, Weihai 264200, China; 202017687@mail.sdu.edu.cn (Y.Z.); 202020923@mail.sdu.edu.cn (E.Z.); 202321244@mail.sdu.edu.cn (H.G.); 202137723@mail.sdu.edu.cn (C.S.); ymx@mail.sdu.edu.cn (M.Y.); 202217717@mail.sdu.edu.cn (M.M.); 202237727@mail.sdu.edu.cn (Y.L.); 202237733@mail.sdu.edu.cn (X.Z.); 2 Shandong Kelun Pharmaceutical Co., Ltd., Binzhou 256600, China

**Keywords:** resistomycin, Wnt signaling pathway, colorectal cancer, apoptosis

## Abstract

Resistomycin is a natural antibiotic related to quinone that has been shown to exhibit robust antitumor activity. To further characterize the mechanistic basis for such activity, human colorectal cancer (CRC) cells were selected as a model to explore the role of Wnt/β-catenin signaling in the ability of resistomycin to induce apoptotic cell death. These analyses revealed that resistomycin was able to suppress β-catenin, TCF4, and GSK-3β expression, together with that of the downstream targets c-Myc and survivin. This coincided with elevated cleaved caspase-3 and Bax protein levels and a decline in Bcl-2 content. When β-catenin was silenced, this further enhanced the ability of resistomycin to induce apoptotic CRC cell death, whereas this apoptotic process was partially ablated when cells were treated using lithium chloride to activate Wnt/β-catenin signaling. Overall, these results support a model wherein resistomycin inhibits Wnt/β-catenin signaling within CRC cells, thereby inducing apoptotic death. Further research may be warranted to better clarify the potential utility of this compound as a candidate drug for use in the treatment of patients suffering from this form of cancer.

## 1. Introduction

Colorectal cancer (CRC) remains the third deadliest form of cancer, and it is particularly common in highly developed nations owing to societal lifestyle and dietary changes in these regions [1,2,3]. CRC often tends to develop at a relatively young age and in a sporadic manner as a consequence of these modern lifestyle practices [4,5,6,7]. Surgery with adjuvant radiotherapy and/or chemotherapy is widely used when treating early-stage CRC patients [8]. Despite advances in the ability to detect this cancer and to treat it with the use of immunotherapeutic agents [9,10], CRC is a highly heterogeneous disease, such that drug resistance is common and many patients face recurrence and consequent treatment failure [11,12]. It is thus vital that new strategies for safely and effectively treating CRC and prolonging the survival of affected patients be identified.

Canonical Wnt/β-catenin signaling is believed to play a central role in CRC oncogenesis [13,14,15]. β-catenin is a transcription factor that plays an essential role in this signaling pathway. Under normal physiological conditions, β-catenin is degraded by a complex that is composed of APC, the scaffold protein Axin, GSK3β, and CK I, with GSK3β and CK I promoting β-catenin phosphorylation, followed by its recognition by E3 ubiquitin ligase (β-TrCP), resulting in its degradation [16,17,18]. Wnt signaling pathway activation can occur upon the binding of secreted Wnt ligands to the Frizzled (FZD) receptors and the co-receptors LRP5/6 [19], the latter of which recruit and disrupt the complex responsible for β-catenin protein degradation, such that it can accumulate within the cytosol [20]. After it accumulates, β-catenin can translocate into the nucleus, wherein it facilitates the transactivation of survivin, c-Myc, and other relevant target genes via interactions with TCF4 [21].

Apoptotic death is a complex process that is modulated by several signaling pathways [22]. Dysregulated apoptosis is a hallmark of a range of diseases, with the suppression of this process, for example, contributing to the aberrant proliferative activity characteristic of cancer development and progression [23]. As such, there is a pressing need to identify compounds capable of modulating apoptotic induction in tumor cells in an effort to overcome therapeutic resistance in cancer patients [24]. Prior studies have explored the link between Wnt/β-catenin signaling and apoptotic activity under particular cellular conditions, targeting this relationship to modulate apoptotic activation using targets such as the Fas and TRAIL receptors, responsible for triggering extrinsic death pathway activity, and Bcl-2/Bax, which are responsible for triggering death via the mitochondrial pathway [25]. Ginkgolide C is reportedly capable of suppressing the proliferation of CRC cells and inducing their apoptotic death via suppressing Wnt/β-catenin pathway activity [26].

**Figure 1:**
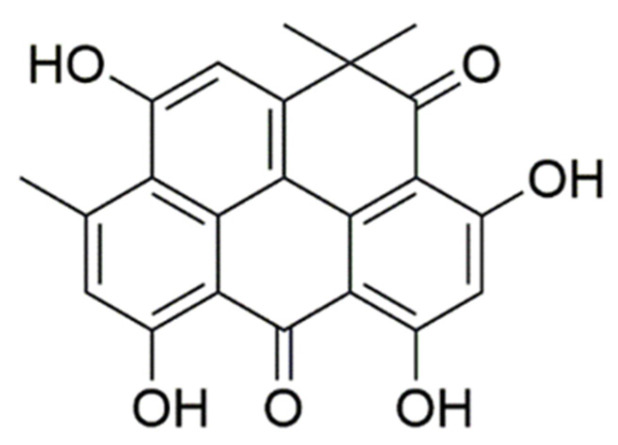
Resistomycin structure.

Resistomycin (Rec) (Figure 1) is a natural antibiotic related to quinone that has been isolated from bioactive Streptomyces species in marine sediment samples collected in the Wehai region of China. Prior reports have demonstrated the robust broad-spectrum antibiotic and antitumor activity of resistomycin in vivo and in vitro [27,28]. Mechanistically, resistomycin has been shown to inhibit the E3 ligase Pellino-1 in triple-negative breast cancer to suppress epithelial–mesenchymal transition (EMT) activity [29], while also inducing HepG2 cell G2/M phase arrest and apoptotic death via the modulation of p38 MAPK signaling activity [30]. The present study was designed to further explore the antitumor effects of resistomycin on CRC cells, ultimately revealing that this novel candidate therapeutic agent may inhibit Wnt/β-catenin pathway activity, suggesting that it may offer utility as a new option for CRC patient treatment.

## 2. Results

### 2.1. Resistomycin Suppresses the Viability of CRC Cells

**Figure 2:**
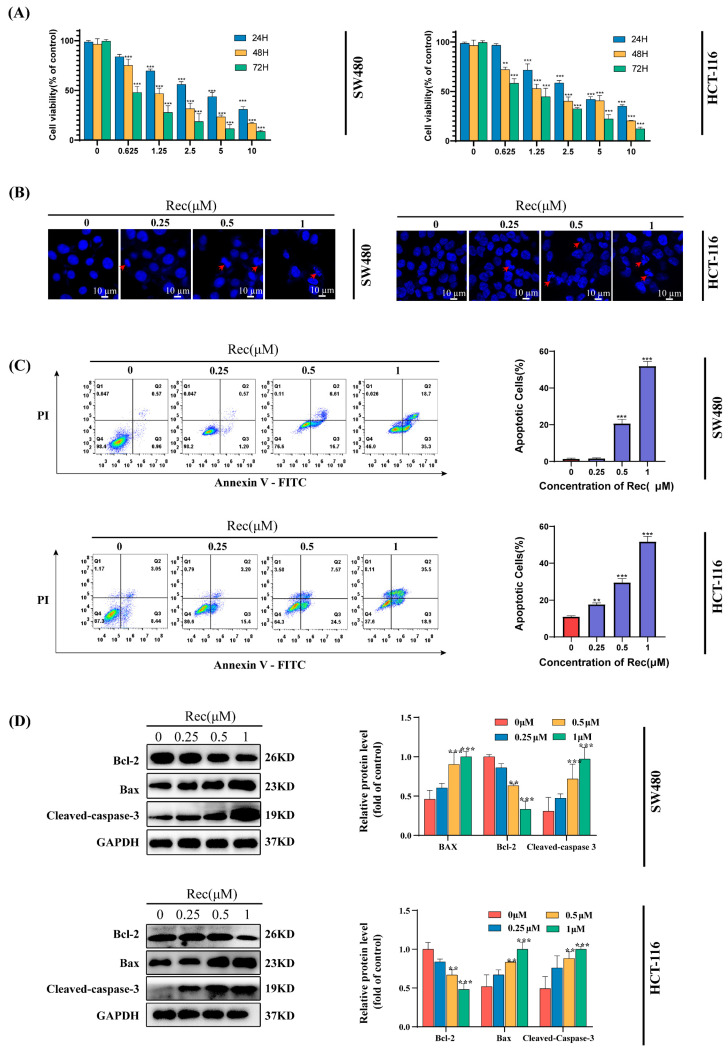
Resistomycin (Rec) inhibits cellular viability and promotes the apoptotic death of CRC cells. (**A**) Resistomycin was found to suppress SW480 and HCT-116 cell viability in a time- and dose- dependent manner. (**B**) Fluorescent images of DAPI-stained SW480 and HCT-116 cells following resistomycin treatment for 24 h. (**C**) Flow cytometry was utilized to analyze the apoptotic death of SW480 and HCT-116 cells following a 24 h treatment with resistomycin. (**D**) Resistomycin-associated changes in Bax, Bcl-2, and cleaved caspase-3 levels were detected using Western immunoblotting. Scale bar: 20 μm. ** *p* < 0.01, *** *p* < 0.001.

Initially, the cytotoxic effects of resistomycin were investigated by using it to treat a range of human CRC cell lines (SW620, HCT-116, HT-29, SW480) and control QSG-7701 liver cells and performing an MTT analysis. This approach revealed that resistomycin strongly inhibited CRC cell proliferation, with a particularly strong impact on the SW480 and HCT-116 cells, while its effects on the normal control cells were less pronounced. Following a 48 h treatment period, the respective resistomycin IC_50_ values for the HT-29, HCT-116, SW620, SW480, and QSG-7701 cell lines were calculated at 3.31 ± 0.41 μmol/L, 1.36 ± 0.26μmol/L, 5.62 ± 0.74 μmol/L, 1.05 ± 0.57 μmol/L, and 15.23 ± 1.44 μmol/L (Table 1). Strikingly, resistomycin was capable of suppressing the proliferation of SW480 and HCT-116 cells in a dose- and time-dependent fashion (Figure 2A), such that these cell lines were selected for further experimental use (the IC_50_ values for HCT-116 and SW480 at 24 h were, respectively, 2.37 ± 0.06 uM and 2.83 ± 0.17 uM).

MTT assays were used to assess resistomycin’s and doxorubicin’s cytotoxic effects on the HT-29, SW480, HCT-116, SW620, and QSG-7701 cells after treatment with various concentrations of these compounds for 48 h. DMSO served as a vehicle control, and the IC_50_ values were calculated. Data are means ± SD (standard deviation) from three independent experiments.

### 2.2. Resistomycin Induces the Apoptotic Death of CRC Cells

Apoptosis is central to the pathogenesis and treatment of cancer, prompting experiments aimed at clarifying whether treatment with resistomycin influences CRC cell apoptotic death. Following treatment with a range of resistomycin concentrations for 24 h, the nuclear morphology was examined via staining with DAPI, revealing pronounced nuclear condensation at lower resistomycin doses (0.25–0.5 μM), whereas fragmentation of the nuclei and apoptotic body formation was observed at 1 μM concentrations (Figure 2B).

Annexin V-FITC and propidium iodide (PI) staining was further employed to gauge the frequency of apoptotic cell death following resistomycin treatment, with cells in quadrants 2 and 3 of the resultant flow cytometry plots being regarded as apoptotic. The frequencies of apoptotic SW480 rose in a dose-dependent manner following resistomycin treatment, (1.53%, 1.77%, 23.31%, 54%) with similar dose-dependent HCT-116 cell death (11.49%, 18.6%, 32.07%, 54.4%) (Figure 2C). These findings provided clear evidence for the ability of resistomycin to induce apoptotic death in both of these CRC cell lines.

To examine the protein level changes underlying resistomycin-induced apoptosis, Western immunoblotting was performed to quantify the cleaved caspase-3, Bax, and Bcl-2 levels. As compared to the control cells, resistomycin promoted Bax and cleaved caspase-3 upregulation, together with Bcl-2 downregulation (Figure 2D). This indicates that resistomycin is capable of triggering the apoptosis of both tested CRC cell lines via the intrinsic death pathway.

### 2.3. Resistomycin Suppresses Wnt/β-Catenin Pathway Activity

**Figure 3:**
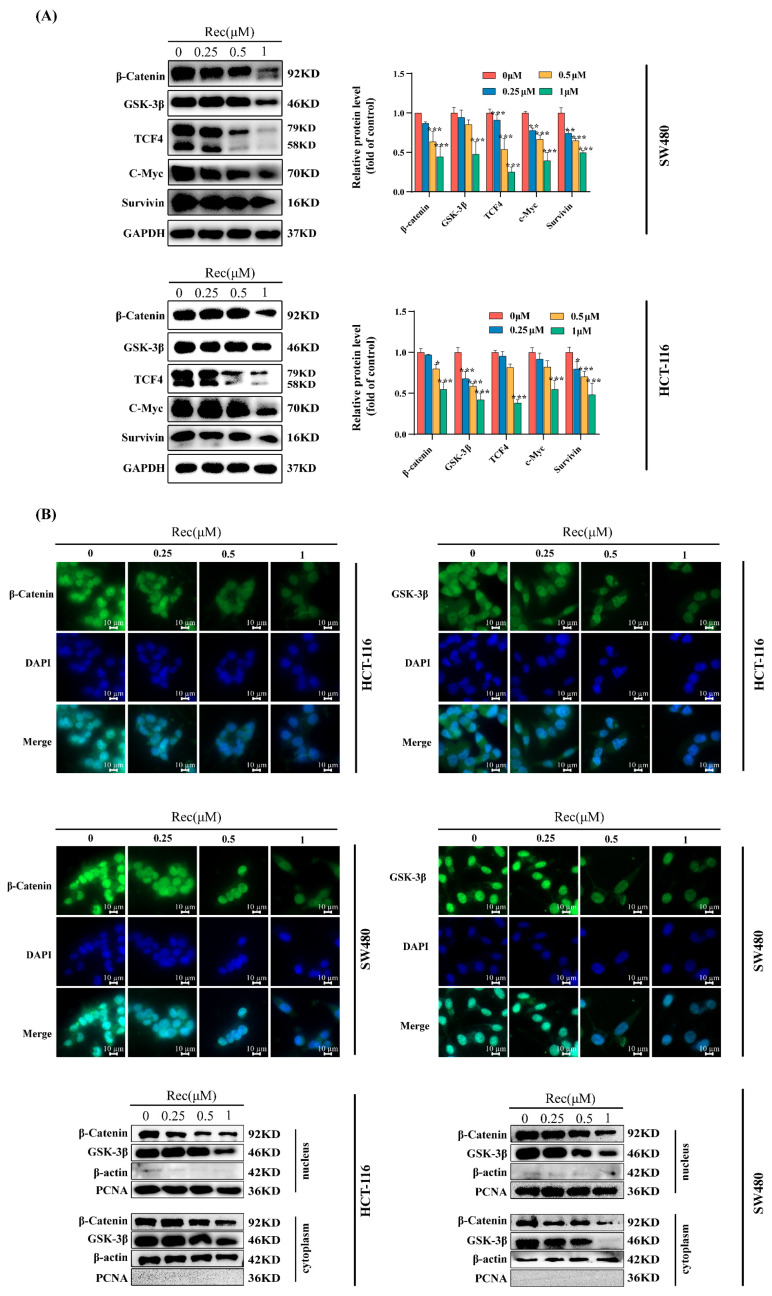
Resistomycin (Rec) suppresses Wnt/β-catenin signaling in CRC cells. (**A**) Resistomycin treatment of SW480 and HCT-116 for 24 h induced changes in β-catenin, TCF4, GSK-3β, and downstream target protein levels were examined via Western immunoblotting. (**B**) After SW480 and HCT-116 were treated with resistomycin for 24 h, β-catenin and GSK-3β (green) were detected via immunofluorescent microscopy, with DAPI (blue) as a nuclear stain, and the intranuclear and extranuclear β-catenin and GSK-3β were assessed via Western immunoblotting. Scale bar: 20 μm. * *p* < 0.05, ** *p* < 0.01, *** *p* < 0.001.

Given that CRC is a highly metastatic form of cancer that often recurs, major barriers to effective treatment remain despite recent therapeutic advances, highlighting a need to clarify how resistomycin impacts metastasis and tumor development. Several reports have highlighted the central role that dysregulated Wnt/β-catenin signaling plays in all aspects of CRC progression. As such, the potential impact of resistomycin on Wnt/β-catenin signaling was next examined by measuring the levels of the β-catenin, GSK-3β, and TCF4 proteins using Western immunoblotting while also assessing the downstream survivin and c-Myc protein targets of this pathway. Resistomycin was found to reduce the β-catenin, GSK-3β, and TCF4 expression in both tested CRC cell lines. Meanwhile, the survivin and c-Myc protein content was correspondingly reduced, which is due to these two proteins acting as downstream of the Wnt/β-catenin pathway and transcription depending on β-catenin translocation into the nucleus, where it interacts with TCF4 (Figure 3A).

β-catenin is an integral component of the Wnt/β-catenin pathway responsible for inducing the expression of downstream target genes, with a tight regulatory relationship between GSK3β and β-catenin having been documented previously. When Wnt signaling is dysregulated, the APC/Axin/GSK-3β complex becomes disrupted such that β-catenin is no longer effectively degraded as a result of LRP5/6 phosphorylation having provided PPPsPxS-motif-binding sites for Axin and GSK-3β [13]. The consequent nuclear accumulation of β-catenin contributes to the upregulation of several oncogenes. Accordingly, immunofluorescent staining and Western immunoblotting were used to assess the localization of β-catenin and GSK-3β in the SW480 and HCT-116 cells following treatment with resistomycin for 24 h, revealing the dose-dependent suppression of nuclear β-catenin accumulation, together with cytosolic GSK-3β expression (Figure 3B). Together, these results suggest that resistomycin is capable of inducing the apoptotic death of CRC cells in vitro by interfering with the Wnt/β-catenin pathway and disrupting the accumulation of β-catenin in the nucleus.

### 2.4. Resistomycin Inhibits Wnt/β-Catenin Signaling to Induce CRC Cell Apoptosis

**Figure 4:**
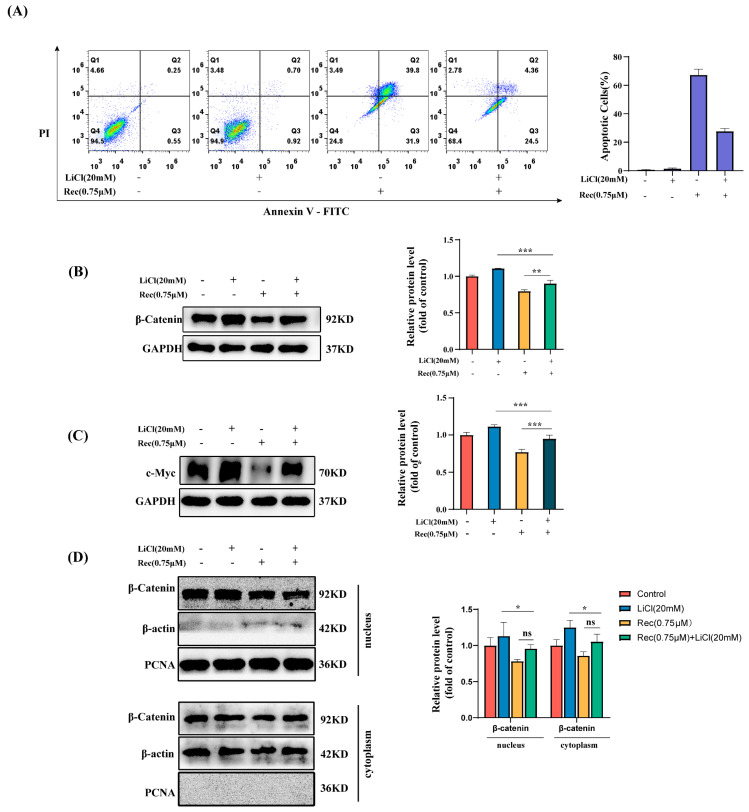
LiCl-mediated Wnt/β-catenin pathway activation partially reverses the effects of resistomycin (Rec). (**A**) HCT-116 cells were treated with LiCl (20 mM) and resistomycin (0.75 M) for 24 h, after which apoptosis of cells was detected via flow cytometry after Annexin V-FITC and PI staining. (**B**,**C**) The HCT-116 treated as in (**A**) and total proteins extracted and the β-catenin and c-Myc assessed via Western immunoblotting. (**D**) The HCT-116 treated as in (**A**) and, respectively, nucleus and cytoplasm proteins extracted; the nuclear and cytoplasmic β-catenin proteins, respectively were assessed using Western immunoblotting. Results are representative of three experiments performed independently. Scale bar: 20 μm. * *p* < 0.05, ** *p* < 0.01, *** *p* < 0.001, ns: not significant.

The suppression of Wnt/β-catenin signaling has previously been reported to contribute to the induction of apoptotic death in CRC cells. For example, Ginkgolide C (GGC) has previously been shown to target this Wnt/β-catenin axis to promote CRC cell apoptosis [26]. Polydatin inhibition of the Wnt/β-catenin pathway in human osteosarcoma cells has been linked to a reduction in the Bax/Bcl-2 ratio, contributing to apoptotic death [31]. Accordingly, the mechanistic link between resistomycin-mediated Wnt/β-catenin pathway inhibition and apoptosis was next tested by utilizing LiCl. The LiCl noncompetitively inhibits GSK3β, then activates Wnt/β-catenin signaling, and disrupts the “APC/Axin/GSK3β/CKI” complex, which leads to the accumulation of β-catenin in the cytoplasm, subsequently causing nuclear translocation [32]. The HCT-116 cells were treated with LiCl (20 mM), resistomycin, or a combination of the two. The β-catenin and c-Myc levels with a combination of resistomycin and LiCl (20 mM) were higher than with resistomycin alone (Figure 4B,C). And, Figure 4D showed that LiCl could reverse the decreased intranuclear and extranuclear β-catenin caused by resistomycin, which accords with the change in the transcription of the downstream target genes of Wnt/β-catenin. Consequently, LiCl reversed the decreases in the β-catenin and c-Myc levels caused by resistomycin, which suggested that resistomycin inhibited HCT-116 cells via the Wnt/β-catenin pathway.

A flow cytometry approach was also used to assess HCT-116 cell treatment with resistomycin and apoptotic death following LiCl treatment. LiCl alone failed to induce apoptosis, while the frequencies of apoptotic death in the resistomycin and combination treatment groups were 70.8% and 28.86%, respectively (Figure 4A). The total numbers of apoptotic cells altered by LiCl and resistomycin treatment as compared to the group that was only treated with resistomycin were lower. This suggests that LiCl was able to partially reverse the apoptosis effects of resistomycin induced in the HCT-116 cells. Moreover, LiCl reversed the decreases in the β-catenin and c-Myc levels caused by resistomycin. Therefore, these data further supported the role of Wnt/β-catenin pathway regulation in the resistomycin-mediated apoptotic death of HCT-116 cells.

### 2.5. β-Catenin Knockdown Inhibits Downstream Signaling and Promotes Apoptotic Death

**Figure 5:**
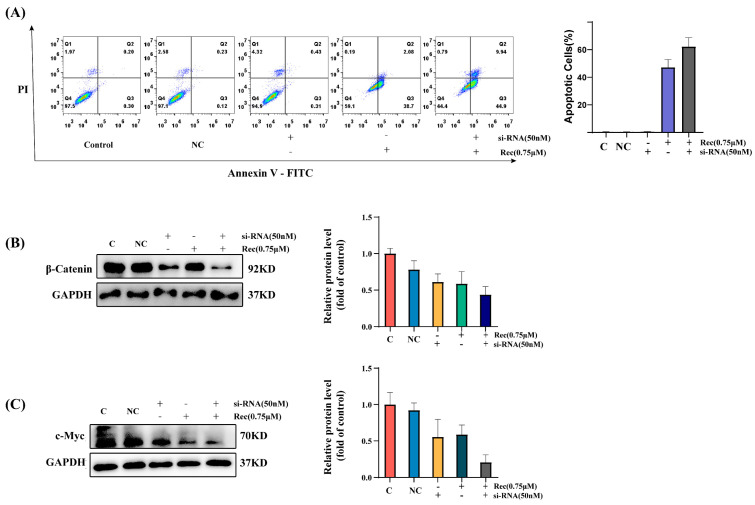
β-catenin pathway silencing induces apoptotic death in CRC cells. (**A**) At 24 h post-transfection with β-catenin-specific or control siRNAs (50 nM), HCT-116 cells were treated for 24 h with resistomycin (Rec), and flow cytometry was used to analyze cells stained using Annexin V-FITC and PI. (**B**,**C**) Western immunoblotting was used to detect β-catenin and c-Myc protein levels after HCT-116 cells were treated as in (**A**). Results are representative of three experiments conducted independently.

To better confirm the role of Wnt/β-catenin pathway signaling activity in the resistomycin-mediated induction of apoptotic death, siRNA-mediated β-catenin silencing was next performed. Western immunoblotting confirmed that the β-catenin and c-Myc levels in cells treated with β-catenin siRNA and resistomycin were significantly lower than those in cells that were only treated with resistomycin (Figure 5B,C). The apoptosis rates were also elevated in the resistomycin + siRNA group as compared to the resistomycin-only group (54.84% vs. 40.87%) (Figure 5A), with more cells at both the early and late stages of apoptosis relative to the control treatment, consistent with β-catenin silencing having contributed to the enhancement of resistomycin-induced apoptotic death.

## 3. Discussion

Apoptosis is the programmed cell death which maintains the healthy survival/death balance in metazoan cells [33]. Cell death via apoptosis also plays a major role in cancer treatment, serving as the main effector function of many anti-cancer therapies [34]. A wide range of antitumor therapeutics have been developed that exert their therapeutic benefits via the targeting of apoptotic-death-related pathways [35,36]. As a quinone-associated antibiotic derived from marine species [37,38,39], resistomycin has been shown to exhibit potent antitumor and antibacterial activity [40,41]. By directly binding to the E3 ligase Pellino-1 in tumor cells, resistomycin has previously been shown to modulate mitochondrial dysfunction, thus impairing the viability of breast cancer and HepG2 cells. Accordingly, resistomycin was herein assessed for its impact on CRC cells, supporting its utility as a potent anti-cancer drug candidate that caused more significant cytotoxic cell death for CRC cell lines as compared to control QSG-7701 hepatocytes, highlighting a potential therapeutic window for this drug. DAPI staining and flow cytometry additionally revealed the ability of resistomycin to induce the dose-dependent apoptotic death of HCT-116 and SW480 cells, while Western immunoblotting revealed a higher pro-apoptotic Bax content in resistomycin-exposed cells, together with a drop in anti-apoptotic Bcl-2 content. Resistomycin can thus promote the apoptosis of CRC cells, with corresponding increases in cleaved caspase-3 levels within both HCT-116 and SW480 cells.

Signaling via the Wnt/β-catenin pathway is integral to the development of many cancers, and is closely tied to the progression of CRC and associated patient prognostic outcomes [42,43], suggesting that efforts to inhibit this pathway may represent a viable antitumor treatment strategy. In the present study, the Western blotting results showed that treatment with resistomycin was sufficient to reduce the protein levels of β-catenin, GSK-3β, TCF4, c-Myc, and survivin within CRC cells. In addition, immunofluorescent staining and Western blotting demonstrated that treatment with resistomycin reduced the levels of GSK-3β and β-catenin in the nucleus and cytoplasm.

Prior studies have demonstrated that Wnt/β-catenin pathway activity can induce or suppress apoptotic activity in particular cellular contexts [44,45,46,47,48]. The alkaloid homoharringtonine can promote HCT-116 cell apoptotic death via the inhibition of Wnt/β-catenin signaling [46], prompting a similar analysis of whether resistomycin was capable of suppressing this pathway and thereby inducing this form of programmed cell death. To test this possibility, LiCl was utilized in the next experiments. LiCl, a GSK-3β inhibitor, destroys the APC/Axin/GSK-3β complex, resulting in the release of β-catenin, which is then free to translocate to the nucleus and initiate the expression of target genes [32]. Western blot results revealed that LiCl treatment reversed the effect of resistomycin inhibition, the β-catenin level both in the total protein, cytoplasm, and nuclear protein, and the Wnt/β-catenin signaling downstream protein c-Myc levels. Moreover, LiCl prominently reversed the resistomycin-induced increase in apoptotic CRC cell death, as assessed using flow cytometry. This thus suggested that resistomycin is capable of driving the apoptosis of CRC cells at least in part via Wnt/β-catenin signaling. In addition, siRNA-mediated β-catenin knockdown prominently intensified the resistomycin-induced apoptosis of CRC cells and inhibition of β-catenin and downstream c-Myc protein levels. These data indicated the resistomycin-induced apoptotic death in CRC cells was caused by the inhibition of the Wnt/β-catenin pathway.

## 4. Materials and Methods

### 4.1. Chemicals and Reagents

DMSO was used to prepare a stock solution of resistomycin (>98% pure) at 1 mmol/L. The antibodies specific to β-catenin, GSK-3β, and TCF-4 were from Cell Signaling Technology (CST, Beverly, MA, USA). The antibodies specific to E-cadherin, N-cadherin, Vimentin, Bcl-2, Bax, cleaved caspase-3, c-Myc, survivin, GAPDH, PCNA, and β-actin and the Nuclear and Cytoplasmic Protein Extraction Kit were from Wanlei Biotechnology (Shenyang, China). The Annexin V-FITC apoptotic detection kit, RNAiMax, and DAPI staining solution were from the Beyotime Institute of Biotechnology (Shanghai, China). The LiCl was from Macklin Biochemical Co., Ltd. (Shanghai, China). The control and β-catenin-specific siRNAs were from Generay Biotech Co., Ltd. (Shanghai, China). All chemicals used herein were of commercial reagent grade.

### 4.2. Cell Culture

The human SW480, HCT-116, and SW620 CRC cell lines and the control liver QSG-7701 cell line were obtained from the Beyotime Institute of Biotechnology (Shanghai, China). The human HT-29 cell line was obtained from Zhong Qiao Xin Zhou Biotechnology Co., Ltd. (Shanghai, China). All the cells were cultured in 37 °C 5% CO_2_ incubators in RPMI-1640 (SW620, HCT-116) or high-glucose DMEM (SW480, HT-29, QSG-7701) (Living Biotechnology Co., Ltd., Beijing, China) containing 10% fetal bovine serum (Gibco, Carlsbad, CA, USA).

### 4.3. MTT Assay

To determine how resistomycin impacts cellular growth, the cells were allowed to adhere to 96-well plates and then treated for 24, 48, or 72 h with various resistomycin compounds, with DMSO (0.1%) being used to treat the control cells. Then, 15 μL of the MTT reagent (5 g/L; Sigma-Aldrich, St. Louis, MO, USA) was incubated for 4 h, followed by the removal of media from each well and the addition of 150 μL DMSO. The absorbance (OD) at 570 nm was then quantified using a microplate reader, and the results were analyzed as follows: (1 − [OD of drug-treated − OD of blank]/[OD of control − OD of blank]) × 100%. The samples were separately analyzed three times, and the IC_50_ values were computed using GraphPad Prism 9.0 (GraphPad, San Diego, CA, USA).

### 4.4. DAPI Staining

The cells were plated on glass coverslips in 24-well plates and allowed to adhere, followed by treatment with varying resistomycin concentrations for 24 h. After rinsing with PBS, these cells were fixed using 4% paraformaldehyde for 15 min, permeabilized using 0.1% Triton X-100 for 20 min, and stained using 4 μg/mL DAPI (Beyotime Biotech, Shanghai, China) for 10 min. After washing them with cold PBS, the coverslips were affixed to the slides using Antifade Mounting Medium, imaged using a fluorescent microscope (Carl Zeiss, Oberkochen, Germany), and processed using the ZEN 3.0 (blue edition) software.

### 4.5. Flow Cytometry

The cells were allowed to adhere to 6-well plates, followed by treatment with various resistomycin treatments for 24 h. The cells were digested using trypsin (Solarbio, Beijing, China) then harvested and centrifuged for 5 min. The supernatant was discarded, rinsed using PBS, and stained using an Annexin V-FITC cell apoptosis detection kit (Bestbio, Shanghai, China) as per the provided directions, followed by analysis using a BD Accuri™ C6 Plus flow cytometer (Becton Dickinson, Franklin Lakes, NJ, USA).

### 4.6. Immunofluorescence

The cells were plated on glass coverslips in 24-well plates and allowed to adhere, followed by treatment with varying resistomycin concentrations for 24 h. After rinsing them with PBS, these cells were fixed using 4% paraformaldehyde for 15 min, permeabilized using 0.1% Triton X-100 for 20 min, blocked for 1 h with 5% BSA, and probed overnight with anti-β-catenin or anti-GSK-3β at 4 °C. After rinsing them with PBS, the cells were treated as per the directions of the Immunol Fluorescence Staining Kit (Wanlei Biotechnology, Shenyang, China). Then, 4 μg/mL of DAPI was applied for 10 min to counterstain the nuclei, and the cell images were captured using an inversed fluorescent microscope (Carl Zeiss, Oberkochen, Germany) and processed using the ZEN 3.0 (blue edition) software.

### 4.7. β-Catenin Silencing

The β-catenin siRNA was validated by Generay Biotech Co, Ltd. (Shanghai, China). A β-catenin-specific siRNA construct was obtained with the following sequences: sense 5′-GAAUACAAUGAUGUAGAATT-3′, antisense 5′-UUCUACAUCAUUUGUAUUCTT-3′. This siRNA (50 nM) or a corresponding negative control construct were transfected into the HCT-116 cells with the 10 uL RNAiMAX transfection reagent per the provided directions, and experimental follow-up was conducted at 24 h post-transfection.

### 4.8. Western Blot Analysis

The cells were allowed to adhere to 6-well plates, followed by treatment with various resistomycin treatments for 24 h. RIPA (Beyotime Biotech, Shanghai, China) was then used to lyse the cells, and the protein content in the harvested lysates was quantified using BCA assay (Beyotime Biotech, Shanghai, China). The intranuclear and extranuclear protein in the HCT-116 and SW480 cells after treatment for 24 h was extracted using the Nuclear and Cytoplasmic Protein Extraction Kit. For each sample, ~30 μg of protein was separated via 8–12% SDS-PAGE and transferred onto PVDF membranes, which were subsequently blocked for 2 h using 5% non-fat milk at 37 °C, rinsed with TBST, and probed overnight with primary antibodies recognizing GAPDH, Bcl-2, Bax, cleaved caspase-3, GSK-3β, β-catenin, survivin, c-Myc, and TCF4 at 4 °C. Secondary anti-mouse IgG or anti-rabbit IgG secondary antibodies (CST, MA, USA) were then used to probe the blots for 1 h at room temperature, followed by protein band detection using an ECL detection kit (ECL^®^, Amersham Biosciences, Little Chalfont, UK).

### 4.9. Statistical Analysis

Data are means ± SEM, and were compared using one-way ANOVA in GraphPad Prism 9.0 (GraphPad, CA, USA). Analyses were performed in triplicate. *p* < 0.05 was selected as the threshold to define significance.

## 5. Conclusions

In conclusion, the present data highlight the ability of resistomycin to induce the in vitro apoptotic death of CRC cells via the suppression of Wnt/β-catenin pathway signaling, suggesting that it may represent a promising novel drug candidate with potential utility for the treatment of patients with CRC. While even low doses of resistomycin were associated with potent antitumor activity in the present study, additional animal model analyses will be essential to clarify the toxicity and specificity of this drug while exploring its potential for drug resistance and combination with other therapeutic agents. Further studies will also be required to better clarify how resistomycin ultimately inhibits Wnt/β-catenin signaling activity at the molecular level. A large number of studies in recent years have focused on clarifying the molecular links between the regulation of Wnt/β-catenin and apoptotic signaling, but the association between these two pathways remains complex owing to the range of receptors and cellular conditions that can shape the related physiological and pathological processes. Given the present data, it appears that resistomycin is capable of suppressing Wnt/β-catenin signaling and caspase-dependent apoptosis. This novel link between resistomycin, apoptosis, and the Wnt/β-catenin axis may provide a novel avenue to the design of new therapeutic agents capable of treating CRC and other devastating forms of cancer.

## Figures and Tables

**Table 1:** Cell-line-specific IC_50_ values for resistomycin and doxorubicin after treatment for 48 h.

Cell Line	IC_50_ (μM/L)	
	Resistomycin	Doxorubicin
HCT-116	1.36 ± 0.26	1.18 ± 0.32
SW480	1.05 ± 0.57	1.01 ± 0.26
SW620	5.62 ± 0.74	1.41 ± 0.93
HT-29	3.31 ± 0.41	1.31 ± 0.39
QSG-7701	15.23 ± 1.44	1.73 ± 0.19

## Data Availability

The data presented in this study are available from the corresponding author upon request.
